# Quality of life, sense of coherence and experiences with three different treatments in patients with psychological distress in primary care: a mixed-methods study

**DOI:** 10.1186/s12906-015-0654-z

**Published:** 2015-04-26

**Authors:** Tina Arvidsdotter, Bertil Marklund, Charles Taft, Sven Kylén

**Affiliations:** Institute of Health and Care Sciences, Sahlgrenska Academy, University of Gothenburg, Gothenburg, Sweden; Research and Development Unit, Primary Health Care Fyrbodal, Vänersborg, Sweden; Department of Primary Health Care, University of Gothenburg, Gothenburg, Sweden

**Keywords:** Acupuncture, Coping, Integrative treatment, Mixed methods, Person-centred care, Primary care, Psychological distress, Quality of life, Salutogenic dialogue, Sense of coherence

## Abstract

**Background:**

Psychological distress is associated with impaired health-related quality of life (HRQL) and poor sense of coherence (SOC). In a previous study, we found that therapeutic acupuncture (TA) and an integrative treatment that combined TA with person-centred approach in a salutogenic dialogue (IT) alleviated anxiety and depression significantly more than conventional treatment (CT) in primary care patients. Here, we report on secondary analyses regarding the HRQL and SOC from that previous pragmatic randomised controlled trial (RCT).

**Method:**

Quantitative and qualitative design. One hundred twenty patients were referred for psychological distress. Quantitative analyses were performed at baseline and after 8 weeks of treatment using the SF-36 mental component summary (MCS), physical component summary (PCS) and the Sense of Coherence-13 (SOC) questionnaires. Qualitative manifest content analyses were based on open-ended questions—*“Have you experienced any changes since the start of the treatment? Will you describe these changes?”*

**Results:**

No baseline differences were found. At 8 weeks, both the IT and TA groups had statistically better scores and greater improvement from baseline on the MCS and SOC than the CT group. The effect sizes were large. No significant differences were found between the IT and TA groups or in relation to the PCS. SOC was highly correlated with the MCS but not with the PCS. Dropout rates were low.

The experiences of the intervention resulted in four categories: *Being heading back; Status quo; Feeling confirmed;* and *Feeling abandoned*, with 13 related subcategories.

**Conclusion:**

IT and TA seem to improve sense of coherence and mental health status in primary care patients with psychological distress, whereas CT appears to be less beneficial. IT and TA appear to be well-accepted and may serve as useful adjunct treatment modalities to standard primary care. Our results are consistent with much of the previous research in highlighting a strong relationship between SOC and mental health status. The written qualitative data described *feeling confirmed* and feeling increased self-efficacy, self-care and faith in the future. Those in the CT group, however, described *feeling abandoned*, missing treatment and experiencing increased emotional and physical problems. More research is needed.

**Trial registration:**

ISRCTN trial number NCT01631500.

## Background

Psychological distress is generally defined as a state of emotional suffering typically characterised by symptoms of depression and anxiety [1,2]. Prolonged stress as an explanation for the underlying causes of psychological distress may be described as an imbalance between demands and the ability to cope with them [3]. There is an ongoing major international problem both for the patients with psychological distress who seek primary health care and for the professional who must detect the patients and give correct and effective interventions [4]. Psychological distress is a widely used concept that is still vague and without specific definition. Attributes of the concept have been variously identified as the inability to cope effectively, changes in emotional status or communication and feelings of discomfort and harm [5]. Undifferentiated combinations of stressors related to either symptoms or events, personality traits and behavioural problems, anxiety and depression symptoms and functional disabilities are also often applied to psychological distress [1,5-7]. Persons with psychological distress (PD) are often living their lives in a state of genuine suffering [2,8]. PD has been identified by Ridner [5] as changes in emotional status in response to a specific stressor or demand that results in harm, either temporary or permanent. Typically, the emotional symptoms of PD are depression (e.g., sadness, hopelessness, loss of interest) and anxiety (e.g., feeling tense, sense of despair, worry about the future), and these are sometimes accompanied by somatic symptoms (e.g., headaches, insomnia) [1,2,5,6,8]. Risk factors for PD include stressful events, lack of valued social roles, high work demands, poor social support, limited decision-making abilities and no strength to foster internal and external resources [6]. This implies that persons with PD do not have the ability to cope with situations that are frustrating or perceived as harmful or threatening [3]. Sense of coherence, SOC, was established by Antonovsky [9] as a holistic salutogenic theory. Three components, comprehensibility (cognitive), manageability (behavioural), and meaningfulness (motivational), form SOC, which reflects a person’s capacity to effectively manage stressful situations and stay well [10]. A review of the literature shows that SOC is negatively and strongly related to anxiety, depression, perceived stressors, burnout, and hopelessness and positively related to locus of control, optimism, self-esteem, and good perceived health and well-being [11]. Patient-centred salutogenic dialogue has been described as promoting a better balance between resources and risks in medicine [12]. Previous studies have shown the important factors between the patient's experience, safety and clinical effectiveness. Health-related quality of life (HRQL) refers to psychological, physical and social functioning in relation to the impact of the disease. Patients with PD have been shown to have significantly worse scores across multiple HRQL domains [13-15] that persist over long periods of time [16].

Persons with PD are frequent visitors in primary health care [17,18] because of symptoms of anxiety and depression, which often co-occur [19,20] and coexist with undifferentiated psychological [21] and somatic disturbances [22,23]. PD goes often undetected in primary care because it is masked by physical complaints [24-26]. Approximately 50% of persons with PD have painful symptoms [27]. Lack of explanation, credibility and effective treatment often lead to frustration for both the health professional and the patient [28-30]. Many persons with PD use complementary and alternative medicine (CAM) mostly after having been treated with conventional medicine but being dissatisfied with the conventional options [31]. In a national survey on the use of CAM therapies to treat anxiety and depression, a total of 57% of those with anxiety and 54% of those with depression reported using CAM therapies; CAM therapies were used more than conventional therapies by persons with anxiety and depression [32]. Acupuncture is a widely used treatment in CAM [33] and can be described as a safe, complex, holistic intervention [34,35]. Systematic reviews show that acupuncture is a promising treatment alternative for reducing PD despite methodological deficiencies [36-40]. Pragmatic trials with acupuncture in primary care showed significant clinical improvements in both the physical and mental components of HRQL in reducing PD [41,42]. Patients have described the experience as having a whole-person effect, changing symptoms, giving them more energy and changing their personal and social identities [43,44].

Reviews of treatments that link physical and psychological symptoms to reduced PD have been highlighted [45,46], and integrative treatments have been requested [47]. We have previously assessed the short-term effects of therapeutic acupuncture, an integrative treatment that combines therapeutic acupuncture with non-directive salutogenic dialogue, and standard care to alleviate psychological distress in primary care patients in a pragmatic randomised controlled study [48]. Our study showed that both the acupuncture and the integrative treatments were effective in reducing anxiety and depression, more effective than standard primary care treatment. The present study reports the results from that study with regard to health-related quality of life and sense of coherence and describes the participants’ treatment experiences. We used a mixed-method design in a primary health care setting.

### Aim

The aim was to evaluate the effects in primary care patients with psychological distress of eight weeks of integrative treatment (IT) vs. therapeutic acupuncture (TA) vs. conventional treatment (CT) on health-related quality of life and sense of coherence and to describe the patients’ experiences with these interventions to gain a deeper understanding of the quantitative responses to reducing psychological distress.

## Method

### Design and setting

This longitudinal, open, pragmatic, randomised controlled study comparing CT, TA and IT was conducted at four primary health care centres in western Sweden during the period 2010–2011. All 10 centres in the region were contacted, and four centres agreed to participate in the study. Two of the centres (one private and one public) were located in small towns (<15,000 inhabitants) and the other two (one private and one public) were in medium-sized towns (15,000–40,000 inhabitants). The resources and clinical approach to treat patients with PD are mainly the same in private and public primary care centres but in order to guard us for minor differences both sorts of centres were chosen. The study was approved by the Regional Ethical Review Board, Gothenburg Sweden (Dnr: 365–08).

### Participants

The population comprised 20- to 55-year-old patients in primary care who were suffering from psychological distress such as worry, sleep disturbances, fatigue, anxiety, depression, headache or somatic pain. Exclusion criteria were full sick leave >2.5 years, pregnancy, cancer, personality disorders, substance or alcohol use disorders and severe depression. In total, 150 patients were referred by healthcare professionals and four were self-referrals; 34 patients failed to meet the inclusion criteria and were excluded; and 120 persons (57 from public and 63 from private primary care centres) were ultimately included in the study. Written information about the study was mailed along with a written consent form which they were asked to return at their next visit. More detailed data on the procedure are reported elsewhere [48]. The patients had a primary diagnosis of depression, anxiety or panic disorders, severe stress, somatic symptoms/pain (including irritable bowel syndrome, fibromyalgia, migraine, fatigue) or sleep disorders. Distribution of the most important baseline data in the three treatment groups, see Table 1. Approximately 80% had secondary diagnoses including one or more of the above primary diagnoses.

**Table 1:** **Baseline data of patient sociodemographic characteristics, primary diagnoses and use of antidepressant medicine**

**Variable**	**IT n = 40**	**TA n = 40**	**CT n = 40**
Sex: female	34 (85%)	32 (80%)	35 (88%)
Mean age	41	41	40
Education: high school	26 (65%)	28 (70%)	23 (58%)
Depression	15 (35,5%)	10 (25%)	12 (30%)
Severe stress	10 (25%)	10 (25%)	10 (25%)
Anxiety/panic disorder	5 (12,5%)	8 (20%)	6 (15%)
Sleep disorders	5 (12,5%)	4 (10%)	5 (13%)
Somatic symptoms/pain	5 (12,5%)	8 (20%)	7 (17%)
Antidepressant medicine	15 (37,5%)	9 (22,5%)	14 (35%)

IT: Integrative treatment, TA: Therapeutic acupuncture, CT: Conventional treatment.

### Interventions

#### Therapeutic acupuncture

Therapeutic acupuncture (TA) [49] was performed once a week for eight consecutive weeks. Each session lasted approximately 45 minutes. Selection of acupuncture points was based on clinical randomised controlled treatments (RCTs) and traditional chinese medicine (TCM) [50] which were presented elsewhere [48]. Between two and 12 needles were used until qi was achieved. While performing TA, the acupuncturist conversed in a free, more unstructured dialogue with the patients about their conditions and suggested various lifestyle changes e.g. diet, exercise, sleep habits and relaxation e.g. breathing exercises for patients to practice at home.

### Integrative treatment

The integrative treatment (IT) combined TA with a person-centred approach [51,52] in a salutogenic dialogue, inspired by Antonovsky’s salutogenic model [9]. The dialogue focused on the patients’ understanding of meaning and resources to help them become aware of and mobilise their strengths and potential for managing their conditions. The dialogue was exploratory and reflected on inner feelings, personal relationships, everyday activities (diet, exercise, relaxation, sleep habits) and existential issues in an atmosphere that aimed to strengthen the therapeutic alliance [53]. The dialogue was structured with goal focus about lifestyle changes. A CD was awarded with 20 minutes guided breathing techniques that was prescribed to practice at home. IT was performed once a week, 60 minutes per session, for eight consecutive weeks.

### Conventional treatment

Patients in the conventional treatment group (CT) were treated according to local practices at each primary care centre. Treatments included watchful waiting and pharmacological and/or psychological or psycho-educational therapies. CT was evaluated after eight weeks of treatment.

All patients who had been prescribed medication were advised about their medication regimens. Patients in the TA and IT groups were asked not to begin psychological treatments or physiotherapy during the study period. In the TA and IT groups, the participants were treated by the same therapist, who had nine years of clinical experience in TCM and years of experience with the salutogenic dialogue inspired by Antonovsky’s sense of coherence theory [9], which is included in nursing education in Sweden.

### Randomisation

**Figure 1:**
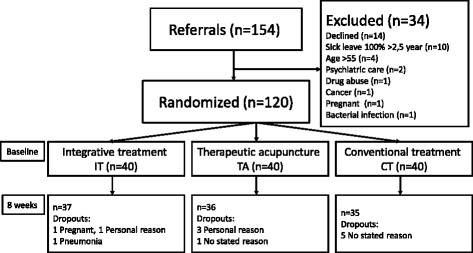
Flowchart of the patients in the study.

After baseline assessments, patients were randomly allocated to one of the three treatment regimens. One hundred and twenty cards printed with the letters A, B or C, designating the treatment group, were prepared and placed in sealed, opaque envelopes by a person with no connection with the study. The envelopes were shuffled and mixed together in a box. A research nurse, blind to the aims of the study and the card coding system, first mixed the envelopes again and then randomly selected envelopes from the box and thereby established the randomisation sequence (Figure 1).

### Data collection and assessment instruments

Assessments were conducted by means of mailed self-rated questionnaires at baseline and after the completion of eight weeks of treatment. Baseline questionnaires were returned at the initial visit and follow-up questionnaires were returned by mail; for more details, see the previous study [48].

The Swedish version of the Short Form-36 (SF-36) version 1.0 [54]. The SF-36 is a 36-item, generic questionnaire that measures self-reported health-related quality of life (HRQL) in eight domains: physical functioning, physical role limitations, bodily pain, vitality, general health, social functioning, emotional role limitations and mental health. Domain scores range from 0–100, with higher scores indicating better HRQL. Domain scores may be aggregated and normalised using a standard algorithm into two summary component scores, the mental component summary (MCS) and the physical component summary (PCS), on which higher scores indicate better HRQL and a value of 50 represents the population norm. For reasons of parsimony, analyses were performed using the MCS and PCS scores.

The 13-item version of the Sense of Coherence (SOC) questionnaire [9]. SOC comprises three components: comprehensibility, manageability and meaningfulness. These concepts are relevant to how people manage different situations. If a person finds a situation comprehensible, manageable and meaningful, the situation becomes less stressful. Items are rated on a seven-point scale, and scores are summed to a range of 13–91. Low SOC scores indicate that the individual may require assistance with finding new strategies to address stressful situations.

### Statistical analyses

Analyses were performed on an intention-to-treat basis. Descriptive statistics were used to characterise socio-demographic, clinical and outcome variables at baseline and follow-up in each treatment group. Baseline between-group differences in gender and education level were assessed with the Chi2 test, and age was evaluated with a one-way ANOVA. Between-group differences on the SOC, SF-36 MCS and SF-36 PCS at baseline and follow-up, as well as changes from baseline, were assessed with the non-parametric omnibus Kruskal-Wallis test, followed by pairwise comparisons with the Mann–Whitney U test. The Wilcoxon signed rank test was used to test for within-group differences between baseline and 8 weeks. Spearman’s rho correlation was used to assess any relationships between the SOC and SF-36 component scores. Non-parametric methods were used because of the skewed distribution and ordinal nature of the SF-36 and SOC data. Scores for missing questionnaires were imputed as the treatment group mean. All tests were two-tailed, and a 5% significance level was used. Bonferroni correction was used to compute adjusted p-values for multiple comparisons. All analyses were conducted using PASW SPSS version 18 (Chicago, Il) [55].

The clinical significance of all changes was assessed using effect sizes. Effect sizes (ES) were calculated to estimate the magnitude of both the within-group changes in SF-36 and SOC values between baseline and 8-week follow-up and the between-group differences at 8-week follow up, as well as score changes. Within-group ES was calculated as the difference between the mean values divided by the standard deviation of score changes. Between-group ES was calculated as the difference between the mean values divided by the pooled standard deviation. ES confidence intervals of 95% were calculated. ES magnitudes were interpreted against the criteria suggested by Cohen: trivial (0 to <0.2), small (≥0.2 to <0.5), moderate (≥0.5 to <0.8) and large (≥0.8) [56].

### Qualitative Data collection

To describe the patients’ experiences, we used open-ended questions to collect written data in addition to the primary outcome questionnaires after the intervention. The open-ended questions were *“Have you experienced any changes since the start of the treatment? Will you describe these changes?”* The statements were analysed using an experience-oriented [57] manifest content analysis [58] in which the literal content of the text was highlighted. The transcribed responses were read based on a comprehensive understanding of the research question. Meaning units were identified, condensed, grouped and interpreted in relation to the study aim. Codes were identified, named and grouped into subcategories. New associations and sentences were sought. The analysis process was a repeated back and forth between the original text and the categories. The text was reviewed and discussed throughout the analysis process between the co-authors for comparison and validation. With this open and critical dialogue, it was possible to develop the final categories through consensus [58].

## Results

### Quantitative

A total of 108 (90%) of the 120 randomised participants completed eight weeks of treatment. Of these, 37 were in the IT group (86% women), 36 in the TA group (83% women) and 35 in the CT group (95% women). (Figure 1). The treatment groups did not differ significantly at baseline with respect to age, gender, education or diagnose (Table 1).

### Between-group comparisons at baseline

No differences between treatment groups were found at baseline on the SOC or the SF-36 including both MCS or PCS (Table 2).

**Table 2:** **Descriptive statistics at baseline and after eight weeks and change between baseline and follow up for integrative treatment (IT), therapeutic acupuncture (TA) and conventional treatment (CT)**

**Outcomes**	**IT (n = 40)**			**TA (n = 40)**			**CT (n = 40)**			**IT-TA-CT**	**IT-CT**	**TA-CT**	**IT-TA**
	**Mean**	**SD**	**Median**	**Mean**	**SD**	**Median**	**Mean**	**SD**	**Median**	**p-value** ^**1**^	**p-value** ^**2**^	**p-value** ^**2**^	**p-value** ^**2**^
SF36 PCS													
Baseline	45,07	11,53	46,88	43,80	10,34	42,77	46,85	9,25	46,49	,384			
8 weeks	46,70	11,10	50,45	47,13	9,30	47,31	46,58	10,51	46,77	,881			
Δ	1,63	9,99	,56	3,33	8,13	1,59	-,27	9,26	,34	,292			
Δ p-value^3^	,333			,026			,968						
SF36 MCS													
Baseline	27,62	13,64	24,47	28,48	13,64	31,27	27,13	12,43	27,60	,896			
8 weeks	46,14	9,35	46,14	43,41	11,90	43,58	32,65	14,08	32,38	**,001**	**,001**	**,001**	,476
Δ	18,52	11,84	18,35	14,93	12,34	11,98	5,52	13,59	5,27	**,001**	**,001**	**,002**	,163
Δ p-value^3^	**,001**			**,001**			**,011**						
SOC													
Baseline	55,38	13,70	56,00	52,15	11,59	54,00	53,08	13,80	51,50	,533			
8 weeks	68,13	9,61	68,01	64,84	10,42	64,95	56,25	13,32	56,41	**,001**	**,001**	**,001**	,195
Δ	12,75	11,35	10,00	12,69	10,85	11,00	3,18	11,52	2,50	**,001**	**,001**	**,001**	,954
Δ p-value^3^	**,001**			**,001**			,091						

SF-36: 36-item Short Form-36 health survey. SF-36 Physical Component Summary (PCS) and Mental Component Summary (MCS) and Sense of Coherence (SOC) ^1^Nonparametric Kruskal-Wallis test, ^2^Mann-Whitney-U test; p-values less than 0.017 (Bonferroni correction of p-values for multiplicity) are shown in bold, p-values for Mann–Whitney-U are shown only where Kruskal-Wallis tests were significant; ^3^ Wilcoxon signed rank test.

Between-group comparisons of baseline scores, 8-week scores and baseline to 8-week change (Δ), as well as within-group change are shown.

### Between-group comparisons at 8-week follow up

Significant differences were found between treatment groups on the SF-36 MCS (p = .001) and the SOC (p = .001) (Table 2). Pairwise comparisons between treatment groups showed that CT had significantly worse MCS and SOC values than did IT and TA (all p = .001). No differences were found between IT and TA on any variable.

**Figure 2:**
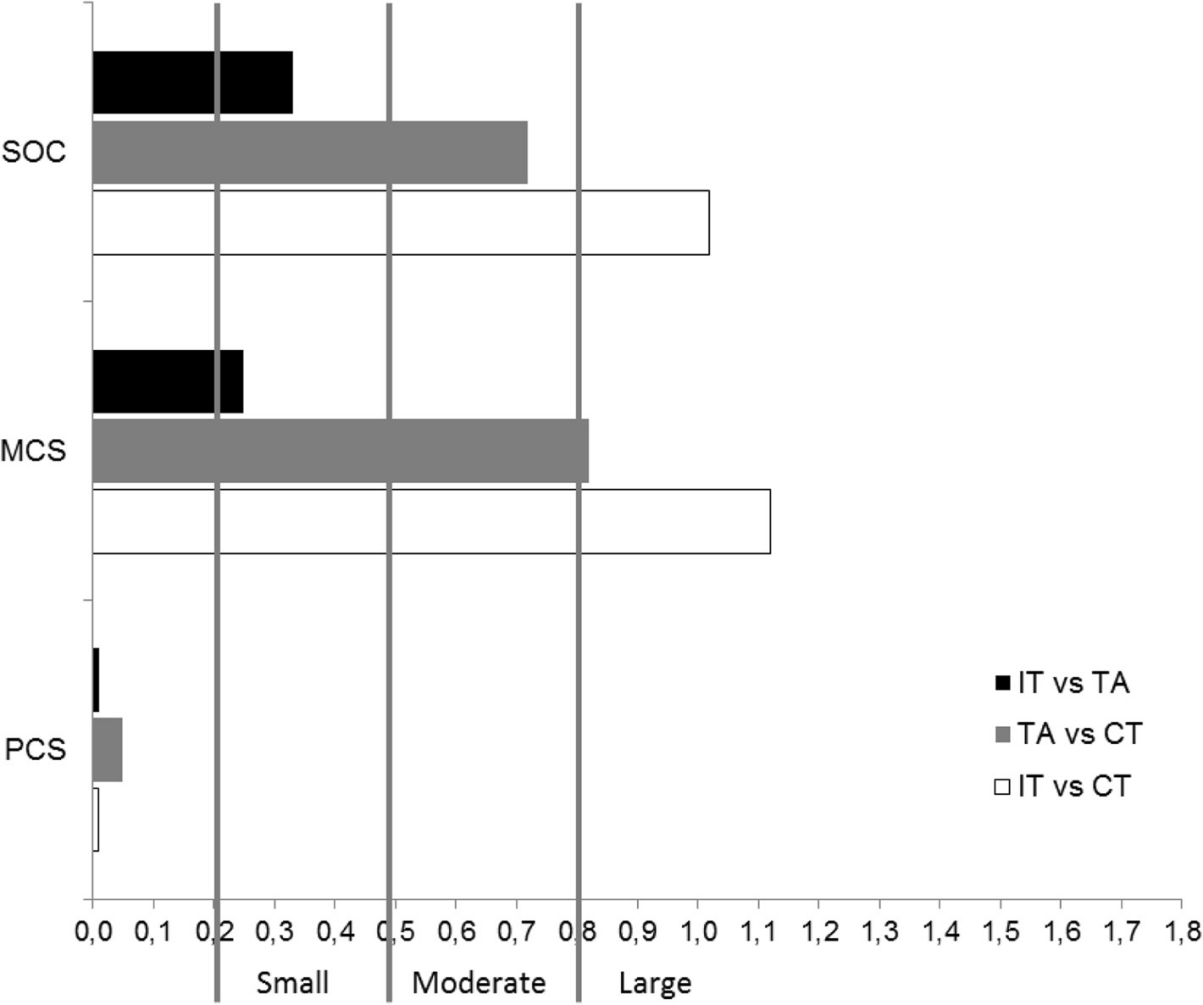
Effect sizes (ES) comparing integrative treatment (IT), therapeutic acupuncture (TA) and conventional treatment (CT) regarding sense of coherence (SOC) and SF-36 physical component summary (PCS) and mental component summary (MCS) scores at 8 weeks. ES reflects the magnitude of the effect on the first group relative to that on the second.

As shown in Figure 2, effect sizes (ES) comparing IT and TA with CT were large in relation to the MCS (ES = 1.12; CI = .64–1.58 and ES = .82; CI = .36–1.27). In relation to SOC, ES was large for IT (ES = 1.02; CI = .55–1.48) and moderate for TA (ES = .72; CI = .26–1.16). Small ESs were observed when IT and TA were compared in relation to the MCS (ES = .25; CI = −.19–.69) and to SOC (ES = .33; CI = −.12–.77). ESs for PCS were all trivial.

### Within-group comparisons of change

**Figure 3:**
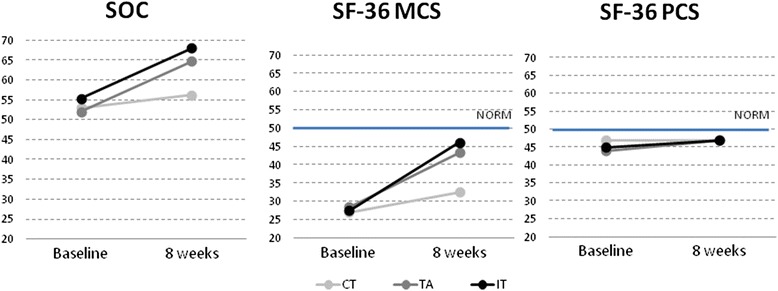
SF-36 physical component summary (PCS) and mental component summary (MCS) and sense of coherence (SOC) scores at baseline and after 8 weeks of integrative treatment, therapeutic acupuncture and conventional treatment.

As illustrated in Figure 3, CT mean scores were largely unchanged from baseline to 8 weeks with respect to the PCS and to SOC, although a slight but significant improvement was observed on the MCS (p = .011). As is also shown in the figure, IT and TA improved on both SOC and the MCS to roughly the same degree. These improvements were significant for both groups (both P = .001). No change was observed in PCS scores from baseline (the change in TA was not significant after correcting for multiplicity). Despite significant improvements from baseline for IT and TA, mean MCS scores remained significantly lower than norm values at 8 weeks.

**Figure 4:**
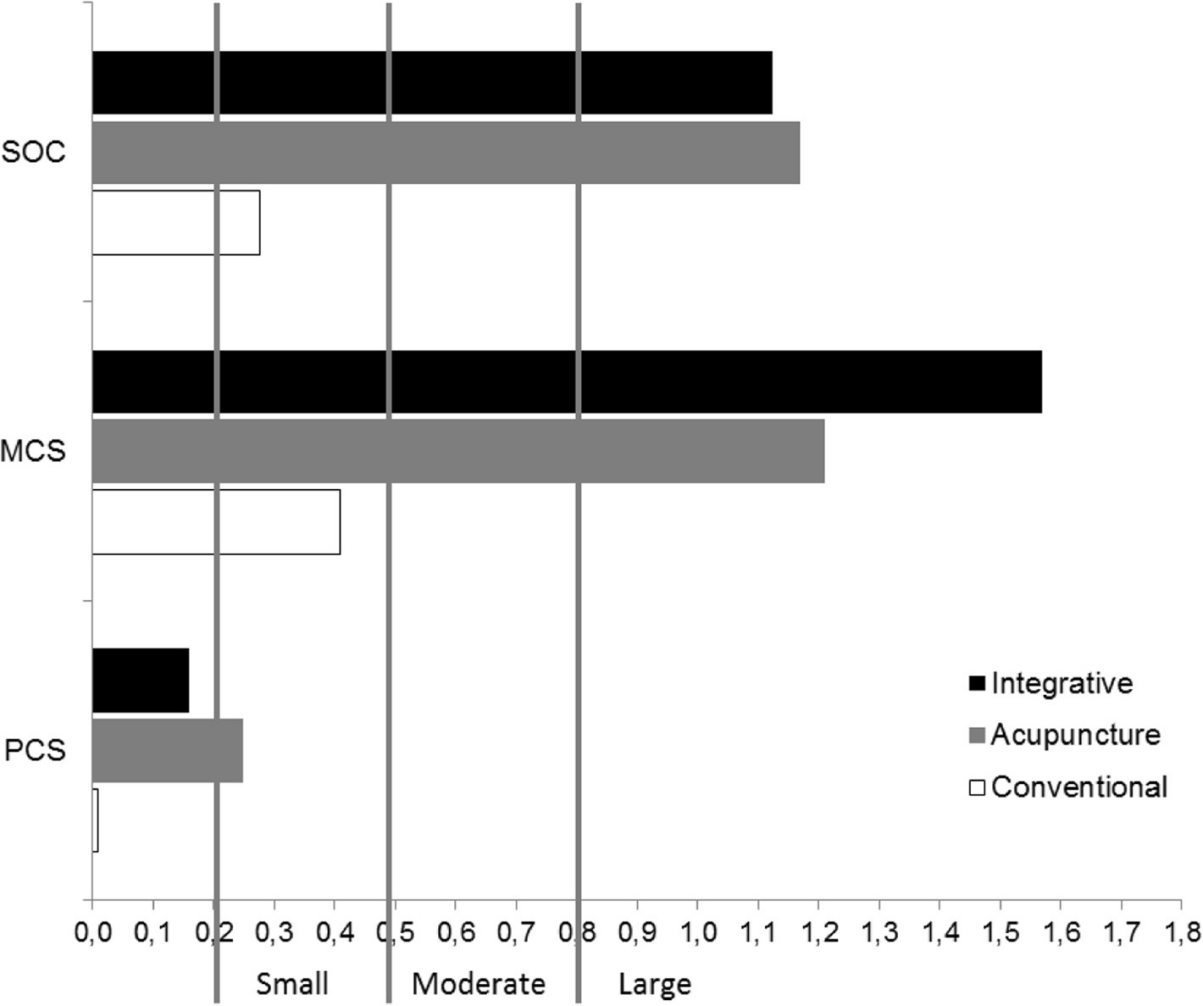
Effect sizes comparing changes between baseline and 8-week follow-up on sense of coherence (SOC) and SF-36 physical component summary (PCS) and mental component summary (MCS) scores for integrative treatment, therapeutic acupuncture and conventional treatment.

As shown in Figure 4, effect sizes (ES) between baseline and 8 weeks were large in both IT and TA in relation to SOC (ES_IT_=1.12, CI_IT_=72-1.68; ES_TA_=1.17, CI_TA_=.71-1.66) and MCS (ES_IT_=1.57, CI_IT_=1.20-2.23; ES_TA_=1.21, CI_TA_=74-1.69). ESs for CT were small for both of these variables (ES_SOC_=.28, CI_SOC_=-.19-.69; ES_MCS_= .41, CI_MCS_=-.04-0.84). ESs for PCS were small to trivial (ES_IT_=.16, CI_IT_=-.29-.59; ES_TA_=.25, CI_TA_=-.21-.67; ES_CT_= .02, CI_CT_=-.46-.42).

### Relationship between Sense of Coherence and HRQL

Spearman’s rho correlations between SOC and the MCS for all patients were large and significant both at baseline (r = .63; p < .001) and at 8 weeks (r = .58; p < .001). In contrast, correlations between SOC and the PCS were small and non-significant both at baseline (r = .20; p = .57) and at 8 weeks (r = .02; p = .92). The sizes of the correlations differed only slightly between groups.

### Dropouts

In total, 3 IT, 4 TA and 5 CT patients dropped out during the study period (Figure 1). Reasons for dropout were pregnancy, personal reasons and pneumonia in the IT group; personal reasons (n = 2) and no stated reason in the TA group; and no stated reason in the CA group.

### Qualitative

The open-ended questions were answered after the intervention by 85% (n = 40) of the IT group, by 84% (n = 40) in the TA group and by 58% (n = 40) in the CT group. The analysis resulted in the following final four categories: *Being heading back; Status quo; Feeling confirmed;* and *Feeling abandoned*, with 13 related subcategories (Table 3).

**Table 3:** **Categories and subcategories during the 8 weeks of treatment**

**Categories**	**Subcategories**	**IT**	**TA**	**CT**
To be heading back	Improved recovery	x	x	-
	Relief from emotional symptoms	x	x	x
	Relief from physical symptoms	x	x	x
	More energy	x	x	x
	Stimulated cognitive ability	x	x	-
	Increased self-awareness	x	x	x
	Changed view on life	x	x	x
	Help to self-help	x	x	x
Status quo	Nothing changed	x	x	x
Feeling confirmed	Being taken seriously	x	x	-
	Pleased with treatment	x	x	-
Feeling abandoned	Lack of treatment	-	-	x
	Increased emotional and physical symptoms	-	-	x

IT: Integrative treatment, TA: Therapeutic acupuncture, CT: Conventional treatment.

### Category I: To be heading back

This category describes the patients’ physical, mental, emotional and cognitive experiences during the eight weeks of treatment. To be heading back emerged as a feeling of patients’ getting in touch with their life situations again and being able to move on.

This category is based on eight subcategories that emerged in the IT, TA and CT groups: *relief from emotional symptoms, relief from physical symptoms, more energy, increased self-awareness, changed views on life* and *increased self-help ability*. Two subcategories emerged in the IT and TA groups: *improved recovery* and *stimulated cognitive ability*.

Improved recovery was described as better sleep, falling asleep more easily, fewer awakenings during the night and, if awakened, less difficulty getting back to sleep, more uninterrupted and deeper sleep and an increased feeling of being rested in the morning. *“The sleep has become much better, which brings that I don’t have the feeling of being constantly tired…”. (30 IT)*

Relief of emotional symptoms emerged as reduced worry, anxiety and depression, greater calm and harmony and feeling happiness and inner joy again that had been absent for a long time.

Relief of physical symptoms emerged as reduced pain, muscle tension, migraine, dizziness, tachycardia and eczema.

More energy emerged as more lust and power to be active and a greater need for social interactions. Stimulated cognitive ability manifested as having less difficulty “finding lost words”, increased memory and concentration and a feeling of being more alert and clear minded. *“Have become more “awake”, I’m cognitive more alert, the eyes radiates alertness…”. (117 IT)*

Increased self-awareness was reflected as increased insight and self-confirmation, as well as increased body awareness, and a changed view of life such that life itself felt brighter was viewed as increased belief in the future. The patients’ attitudes towards pain changed, and the intensity of pain decreased. Increased acceptance made it easier to address misfortunes and difficult situations. There were fewer musts and demands, which made it easier for the patients to impose limits. Increased self-help emerged as part of the patients’ own relaxation, breathing or physical activity exercises. *“I can control my thoughts by breathing techniques, thoughts that earlier took me down. I’ve been helped to lead my thoughts in other directions…”. (31 IT)*

### Category II: Status quo

The category status quo implies that *nothing changed*—treatments had no effect on the patient’s condition. Physical and emotional symptoms neither improved nor worsened, and the patients doubted that their health would be improved. *“It varies very up and down, so it’s hard to answer”. (91 TA) “Has been slightly more positive but now again a little worse, so right now about the same as the start”. (77 CT) “… no major change”. (90 IT)*

### Category III: Feeling confirmed

Feeling confirmed only appeared in the IT and TA groups. This category contained the two subcategories *being taken seriously* and *being pleased with treatment*. The feeling of being taken seriously was reflected in the sense that someone was listening to the patients and considered them as individuals rather than merely symptoms. The salutogenic dialogue was experienced as rewarding, and the treatments were regarded as valuable both for the patients’ present situations and for the future. A number of participants expressed great gratitude for the treatments. *“Eight weeks of treatment made wonders. I wish it could continue!!”. (25 IT) “To me it’s been an amazing difference… who has lifted thoughts with me so I have found right in life”. (21 TA)*

Being pleased with the treatment referred to statements that the patient had been offered a non-pharmacological treatment as a complement to the physician’s intervention. *“This treatment should be available for everyone with pain, if they want it…”. (14 IT)*

### Category IV: Feeling abandoned

Feelings of being abandoned only appeared in the CT group. This category comprised two subcategories: *lack of treatment* and *increased emotional and physical symptoms*. Lack of treatment, pharmacological treatment as the only option, long waits to see professionals, and lack of ongoing rehabilitation resulted in deteriorated health conditions and increased emotional and physical symptoms. *“I became more ill, increased worry, depression and higher dose of medicine. No professional conversations, long waiting time and no treatment led to increased health problems so I was forced to seek acute help at the psychiatric unit. The medicines were changed and I’m connected to the mobile emergency team”. (103 CT)*

## Discussion

This pragmatic, randomised, controlled study showed that both integrative treatment and therapeutic acupuncture significantly, both statistically and clinically, increased coping ability and health-related quality of life in primary care patients with psychological distress; however, no differences were found between these groups. In the CT group, primary care treatment showed only a small and clinically non-significant effect. Dropouts were low in all three treatment groups: only 3 patients in IT, 4 in TA and 5 in CT had dropped out at eight weeks of treatment. Missing data scores for these patients were imputed as the group mean for each respective treatment group [54].

In the IT and TA groups, HRQL showed significant clinical improvement on the SF-36 MCS components of vitality and of social, emotional and mental well-being. One explanation may be that there was a synergy effect of the either structured (IT) or unstructured (TA) dialogue that was integrated with acupuncture in the IT and TA groups. Acupuncture promotes relaxation, and if unexpected thoughts or feelings arise, these can be processed in the moment, decreasing anxiety and depression [41,42]. When the “crippling” anxiety and depressive symptoms are reduced, life feels better, quality of life increases and life becomes more manageable [43,44]. Patients are therefore particularly susceptible to structured or unstructured dialogue that stimulates the healing processes that have already begun [51]. Dialogue encourages processing to identify and manage thoughts and emotions from the past and in the present and the future [9]. It stimulates cognitive ability [45,46] and thus the capacity [12,59] to understand and recognise the difference between internal and external resources, and it gives the person a new and more structured way of thinking and acting [9,51].

There are no boundaries for a normal SOC score; Antonovsky [9] only discussed strong and weak SOC, not upper or lower score limits. SOC seems to have an impact on quality of life, a state of well-being with both objective and subjective dimensions. A previous study [11] showed that SOC and quality of life, in particular, mental health, accompanied each other: the higher the SOC, the better the mental health, which was confirmed by our results for both IT and TA, which showed large ESs. We did not locate any studies on using acupuncture to reduce psychological distress based on SOC outcomes.

Following treatment, patients in all three groups described feeling that they were *heading back*, with the exception of the subcategory *stimulated cognitive ability*, which the CT group did not describe. This could be the result of the structured vs. unstructured dialogue in IT and TA. The salutogenic effects of talk therapy on people with mental health problems and their life demands showed significantly improved SOC scores compared with those of the control group [60]. With improved coping strategies, the symptoms of psychological distress can reduce [1,3,9,61]. SOC appears to protect against various aspects of mental ill health, as do similar concepts, e.g., self-efficacy, hardiness, emotional stability and social support [62]. Active elements of increased clinical effectiveness have been presented as: being taken seriously; being listened to; talking about problems; and increased self-efficacy and self-care [31,34,45,63,64]. This may be a reflection of a positive therapeutic alliance as a result of a person-centred holistic approach that leads to a more active patient [53,65].

The combination of acupuncture treatments with both structured and unstructured salutogenic dialogue may explain our SOC results, specifically, the significant and clinically relevant improvement in large IT ES compared with the small CT ES. In addition, the TA group had a large ES, and the results showed no significant effect between the IT and TA groups. One explanation could be that even the TA group received dialogue (unstructured) and that there was some contamination between groups because the same therapist was used for both groups.

The feeling of *being headed back* through the subcategory *improved recovery* was described only in the IT and TA groups. This finding, combined with the fact that only the CT group experienced *feeling abandoned, a lack of treatment* and *increased emotional and physical symptoms*, could explain the increased HRQL in the IT and TA groups compared with the CT group. Research has shown that patient experience and clinical effectiveness are associated [64], which could explain the feelings of resignation and not feeling confirmed that result in poor treatment outcomes, such as occurred with the CT group. In the present study, the therapist used a salutogenic approach, with experience and training as a nurse, and the acupuncturist had had elementary education in psychotherapy. Could it be that a mix of education and training can provide interventions for individuals in psychological distress in the primary care setting? There is a need for further research.

The effects of acupuncture on reducing psychological distress have been demonstrated in previous studies [36,38-40,66], but few have been in the context of primary care [41,42] and fewer still have looked at integrating a clinical nurse acupuncturist into integrative treatment in a primary care setting [48]. Acupuncture has been seen as a complex but tolerable non-pharmacological intervention [34]. The effects of acupuncture on psychological distress are not fully understood, but according to studies [67,68], these symptoms generally depend on disorders of the autonomic cardiac control system, with excess sympathetic and reduced parasympathetic regulation. General mental health improvement depends on multiple factors, including acupuncture’s anxiety-relieving effects [41,44], which are of the greatest value [67,68]; moreover, the clinical effects on the autonomic nervous system have been confirmed [69,70]. The relationship between the autonomic cardiac control system and acupuncture could help to explain the mechanism of acupuncture’s effects on treating psychological distress [71], and in the relationship between SOC and cardiac autonomic activity, a higher SOC score was positively correlated with parasympathetic regulation [72].

It is also notable that the experience of *feeling confirmed* only emerged in the IT and TA groups. Confirmation, *to be taken seriously*, to be listened to, to be allowed to talk about problems and to gain access to holistic treatment could all have helped participants experience an increased ability to cope with their situations, and this is supported by previous research [31,34,44,63]. *Feeling pleased with treatment* was described by participants who had access to non-pharmacological treatments. Expanding the GP’s agenda [46], can lead to more active patients with increased self-efficacy and self-care and improved clinical outcomes, as has been confirmed in previous studies [45,64]. Participants in the IT and TA groups felt *pleased with treatment*, but not those in the CT group. This may reflect the positive therapeutic alliance that results from a person-centred (holistic) approach; this effect has also previously been confirmed [53,65], and it may provide another explanation for the difference between the IT and TA groups and the CT group. The patient sample was heterogeneous with respect to diagnosis, which reflects the diversity of mental and somatic health problems found in primary care. It is important to note, however, that patients were not necessarily referred for treatment for their primary diagnoses but for suspected underlying psychological distress. Hence, patients with, for example, medically unexplained syndromes, such as IBS and fibromyalgia, were not referred for treatment for their somatic symptoms but for the distress associated with their conditions. In this respect, the sample was homogeneous.

## Conclusion

This pragmatic randomised trial showed that both IT and TA significantly, both statistically and clinically, increased coping ability and health-related quality of life in primary care patients with psychological distress, but no significant differences were found between the two groups. CT in primary care treatment showed only a small and clinically non-significant effect. The written qualitative data provide a deeper understanding of the IT and TA groups’ quantitative responses, in which they described *feeling confirmed*, feeling self-efficacy, and feeling increased self-care and faith for the future. The CT group, in contrast, described experiences of *feeling abandoned*, missing treatment and experiencing increased emotional and physical problems. Our secondary analysis confirmed our previous results [48] regarding reducing anxiety and depression in patients with psychological distress. IT and TA appeared to increase self-care and self-efficacy and were well accepted. These findings confirmed those from previous studies that explored the relationship between the patient’s experience, safety and clinical effectiveness [64]. The clinical effectiveness of integrative treatment could be a nurse-sensitive primary care outcome that reduces psychological distress. Although mental health problems are prevalent, their detection, diagnosis and treatment are inadequate in the primary care setting [4]. Our results indicate that integrative treatment may be a useful and acceptable adjunct regimen for patients identified with symptoms of psychological distress in such settings, and this is in line with the WHO’s recommendations [73] for incorporating CAM methods into health care. Appropriate evaluation parameters need to be developed and included to evaluate the success of interventions in a person-centred salutogenic system [74].

We know that integrative treatment and therapeutic acupuncture in this study have given effects. However we do not know which parts of the intervention which have had effects; salutogenic dialogue (lifestyle changes and relaxation) or acupuncture. More research is needed, however, to examine if the integrative treatment offers additional clinical benefit over acupuncture alone.
